# Ready for Take-off? – Gestaltung und Wahrnehmung von Reiseimpfberatungschatbots

**DOI:** 10.1365/s40702-022-00927-z

**Published:** 2022-11-02

**Authors:** R. Stefan Greulich, Nico Pietrantoni, Fabian Hildebrandt, Tomy Hommel, Stefan Morana, Alfred Benedikt Brendel

**Affiliations:** 1 Technische Universität Dresden, Dresden, Deutschland; 2 Universität des Saarlandes, Saarbrücken, Deutschland

**Keywords:** Digital Health, Conversational Agent, Impfung, Anthropomorphismus., Digital Health, Conversational Agent, Vaccination, Anthropomorphisms.

## Abstract

Der Einsatz von Sprachassistenten (Alltagsbeispiele sind Alexa von Amazon, Siri von Apple oder der Chatbot von Hellofresh) hat auch im Gesundheitswesen Einzug gehalten. Ein aktuelles Beispiel ist der WhatsApp Chatbot der WHO, welcher Nutzer:innen über COVID-19 aufklärt. Sprachassistenten haben die Fähigkeit, Patienten und Patientinnen orts- und zeitunabhängig aufzuklären, wodurch Mitarbeiter:innen entlastet werden. Jedoch gibt es neben den technischen (u. a. Entwicklung und Optimierung der Algorithmen für die Spracherkennung) auch Herausforderungen in der Mensch-Chatbot-Interaktion. In dieser Studie wird untersucht, welche Rolle die menschenähnliche Gestaltung (u. a. menschlicher Name, Begrüßung, menschlicher Avatar) eines Reiseimpfberatungschatbots auf dessen Wahrnehmung durch Nutzer:innen hat. Spezifisch geht es darum zu verstehen, ob und wie sich Anthropomorphismus (die Wahrnehmung von Menschlichkeit und sozialer Präsenz in Objekten, Tieren und Maschinen) auf die wahrgenommene Vertrauenswürdigkeit und letztendliche Zufriedenheit mit dem Service auswirkt. In einem Online-Experiment mit 78 Teilnehmer:innen, wurden zwei Chatbot-Gestaltungen (mit menschenähnlichen Gestaltungselementen vs. ohne diese Elemente) verglichen. Die Ergebnisse zeigen, dass die wahrgenommene soziale Präsenz signifikant die wahrgenommene Vertrauenswürdigkeit und die Zufriedenheit mit dem Service des Chatbots erhöhen. Somit ist die Implikation, dass bei der Reiseimpfberatung und ähnlichen Beratungsprozessen eine menschliche Gestaltung der Sprachassistenten zu empfehlen ist.

## Abstract (en)

The rise of digital speech-based assistants (e.g., Amazon’s Alexa, Apple’s Siri, or Hellofresh’s chatbot) gained increased popularity and has also found its way into healthcare. A current example is WHO’s WhatsApp chatbot that informs users about COVID-19. Such assistants can educate patients, independent of location and time, which is an incredible benefit for patients. However, in addition to existing technical challenges (including the development and optimization of speech recognition algorithms), there are also challenges in human-chatbot-interactions. This study investigates the role of a human-like design (including human name, greeting, and human avatar) of a travel vaccination advice chatbot on the perception of its users. Specifically, we aim to understand whether and how anthropomorphism (the perception of humanness and social presence in objects, animals, and machines) affects perceived trustworthiness and ultimately service satisfaction. In an online experiment with 78 participants, two chatbot designs (with human-like design elements vs. without these elements) were compared. The results show that perceived social presence significantly increases perceived trustworthiness and service satisfaction. Thus, we recommend that practitioners implement a human-like design travel vaccination counseling and similar counseling processes via chatbots.

## Einleitung

Reisen nehmen eine immer größere Stellung in unserer globalisierten Welt ein. Eine Auslandsreise stellt allerdings auch ein gesundheitliches Risiko dar. Viele dieser Risiken können durch einfache Handlungen wie medizinische Aufklärung und Impfungen minimiert werden. Dies widerspiegelt sich in der Tatsache, dass viele erkrankte Reisende keine reisemedizinische Beratung gesucht hatten (Warne et al. 2014). Dementsprechend besteht ein starker Aufklärungsbedarf bzgl. Reiseerkrankungen, um entsprechende krankheitsbedingte betriebs- und volkswirtschaftliche Ausfälle zu verringern. Vor allem sind niedrigschwellige Angebote wichtig, welche Reisende mit geringem Aufwand wahrnehmen können. Ein vielversprechender Ansatz ist der Einsatz von Sprachassistenten.

Dabei werden Sprachassistenten als technische Dialogsysteme verstanden, welche auf Anfragen der Nutzenden reagieren und Fragen oder Befehle ausführen, wie z. B. „Wie wird das Wetter morgen?“ oder „Bitte spiele meine Lieblingssongs“. Alltagsbeispiele sind Siri von Apple, Alexa von Amazon, Cortana von Microsoft und Google Home bzw. Assistent. Vor allem im medizinischen Kontext können Sprachassistenten den Einsatzbereich erweitern und nicht nur bei der Betreuung von älteren Menschen unterstützen, sondern auch im Zuge von Erinnerungsaufgaben Patienten und Patientinnen an ihre Medikamentation aufmerksam machen und so lebensrettende Aufgaben übernehmen.

Diese Art der Assistenten, worunter auch Chatbots fallen, werden immer stärker in der medizinischen Aufklärung genutzt, um eine breite Masse zu erreichen, wie z. B. zur COVID-19-Aufklärung (World-Health-Organization 2021). Dies hat sowohl für die Nutzenden als auch für den Anbieter viele Vorteile. So ist die Information rund um die Uhr abrufbar und bindet keine Fachkräfte. Zusätzlich ist die Hemmschwelle der Nutzer:innen geringer, da diese nur eine Website aufrufen oder im Messenger ihrer Wahl kommunizieren müssen, anstatt sich telefonisch einen Arzttermin zu buchen und diesen wahrzunehmen.

In der medizinischen Aufklärung spielt neben den oben genannten Punkten auch das Vertrauen in die Quelle eine große Rolle (Fiscella et al. 2004). Dies ist im direkten Kontakt zum Arzt oder Ärztin gegeben, da Ärzte und Ärztinnen für gewöhnlich als sehr gut ausgebildet und vertrauensvoll wahrgenommen werden. Dementsprechend ist es wichtig, dieses Vertrauen auch in der Interaktion mit dem Chatbot aufzubauen. In der Human-Computer-Interface (HCI) Forschung sind als Treiber für das Vertrauen die Fähigkeiten des Chatbots und die menschenähnliche Gestaltung identifiziert worden (Toader et al. 2020). Dieser Zusammenhang wurde im Gesundheitskontext und reisemedizinische Aufklärung nach unserem Wissen bisher noch nicht untersucht. Entsprechend wird in diesem Artikel folgende Forschungsfrage adressiert:

### FF

Welche Auswirkung hat die wahrgenommene Menschlichkeit auf das Vertrauen und die Zufriedenheit von Nutzern und Nutzerinnen von Chatbots für die Reiseimpfungsberatung?

Dafür führten wir ein Online-Experiment mit 78 Teilnehmern und Teilnehmerinnen durch und erfassten den Einfluss von menschenähnlichen Gestaltungselementen auf wahrgenommene Menschlichkeit, Vertrauen und Service Zufriedenheit. Zusammenfassend konnte anhand der Daten festgestellt werden, dass menschenähnliche Gestaltungselemente die soziale Präsenz in diesem Kontext erhöht. Diese Wahrnehmung treibt über die Faktoren Fähigkeit und Integrität das Vertrauen der Nutzer:innen, welches wiederum die Service Zufriedenheit erhöht. Abschließend werden die Implikationen dieser Ergebnisse für den praktischen Einsatz von Chatbots im Gesundheitssektor betrachtet.

## Forschungskontext

### Digitale Sprachassistenten im Gesundheitswesen

Im Gesundheitswesen gilt ELIZA als einer der ersten Chatbots und wurde im Jahr 1966 entwickelt, um einen Therapeuten nachzuahmen (Weizenbaum 1966). Seitdem wurden Chatbots kontinuierlich weiterentwickelt und haben den Einsatzbereich innerhalb des Gesundheitswesens erweitert, u. a. zum Thema telemedizinische Krankheitsberatung, Medikamenteneinhaltung und psychiatrische Beratung. Dabei können Chatbots den Patienten und Patientinnen eine besondere User Experience sowie Kunden- und Patientenerfahrung bieten, die nicht nur das Befüllen statischer Informationsformulare übernehmen. Chatbots können als persönliche Assistenten eingesetzt werden, die unabhängig von Zeit und Ort z. B. an die richtige Medikamentation erinnern, medizinische Informationen bereithalten und erste Abschätzungen bzgl. Krankheiten abgeben können, wie im Falle eines Covid-19-Chatbots, der anhand diverser Fragen beurteilt, ob eine ärztliche Konsultation notwendig sei (Brendel et al. 2022).

Darüber hinaus haben sich Chatbots während der COVID-19-Pandemie als innovatives Instrument herausgestellt, um Wissen über präventive Handlungsmaßnahmen zum Infektionsgeschehen zu vermitteln als auch zur Bekämpfung von Fake News (Judson et al. 2020). So wurde beispielsweise der Chatbot Clara vom Center for Disease Control and Prevention als öffentliches Selbstkontrollinstrument eingeführt, das verschiedene Fragen zum individuellen Impfstatus und zu Gesundheitssymptomen stellt und anschließend Empfehlungen abgibt (z. B. bleiben Sie zu Hause und machen Sie einen Selbsttest) (CDC 2020). Allerdings hat sich auch im Gesundheitsbereiche gezeigt, dass die Gestaltung des Chatbots, insbesondere eine menschenähnliche Gestaltung ausschlaggebend für die Wirkung auf die Nutzer:innen hat (Brendel et al. 2022).

### Wirkung menschenähnlicher Gestaltungselemente in Chatbots

Die Neigung, Objekte menschenähnlichen Eigenschaften zuzuschreiben, ist eine tief verwurzelte Voreingenommenheit und führt dazu, dass Objekte wie Zeichentrickfiguren (z. B. Bugs Bunny) mit menschlichen Eigenschaften assoziiert werden (Epley et al. 2007). Dieser Effekt ist auch bei Benutzer:innen von Computern beobachtbar, welche mit einer menschenähnlichen Hard- und/oder Software sozial und kommunikativ interagieren. Diese schreiben dem Computer unbewusst ein gewisses Maß an Menschlichkeit und sozialer Präsenz zu, obwohl sie wissen, dass es sich nicht um ein menschliches Wesen handelt. Dieser Effekt ist unter dem CASA-Paradigma (Computers Are Social Actors) bekannt (Nass et al. 1994) und wird durch die Theorie der sozialen Reaktion erklärt, wonach Menschen automatisch auf soziale Signale reagieren, auch wenn dies nur vom Computer simuliert werden (Nass und Moon 2000). Diese Signale sorgen dafür, dass die Situation nicht wie eine Interaktion mit einer Maschine, sondern mit einem anderen Menschen behandelt wird.

Dabei hängen die Intensität und der Umfang dieser Reaktionen vom wahrgenommenen Grad der Menschlichkeit und der sozialen Präsenz des Chatbots ab. Studien zeigen, dass Nutzer:innen verschiedene affektive, kognitive und verhaltensbezogene Reaktionen auf das menschenähnliche Design eines Chatbots zeigen, z. B. erhöhte Begeisterung, Überzeugungskraft und attribuierte Vertrauenswürdigkeit (Diederich et al. 2020).

## Modell und Hypothesenherleitung

**Abb. 1:**
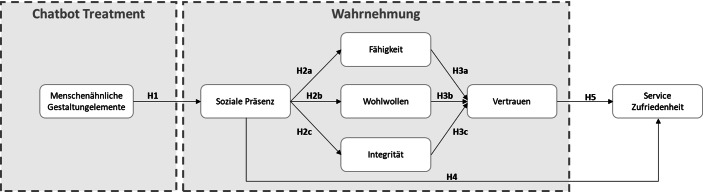
Forschungsmodell

Unsere Studie untersucht, wie Chatbots im Zusammenhang mit reisemedizinischer Beratung gestaltet werden sollten, um größtmögliche Auswirkungen auf die Service Zufriedenheit und Kundenbindung zu erreichen. Auf Basis des Anthropomorphismus-Bias untersuchen wir, wie die wahrgenommene Menschlichkeit das Vertrauen und die Service Zufriedenheit des Chatbots beeinflusst. Unser Forschungsmodell ist in Abb. 1. zusammengefasst und im folgendem werden die angegebenen Zusammenhänge aus der Literatur hergeleitet.

Die Bedeutung einer individuellen und besonderen Customer Experience spielt in vielen Branchen eine bedeutende Rolle, wenn es gilt, den Kunden und die Kundeninnen vom eigenen Produkt oder Service zu überzeugen. Der direkte Kontakt ist dabei elementar, um die Service Zufriedenheit und Kundenbindung zu beeinflussen. In Mensch-Computer-Interaktionen bezieht sich die soziale Präsenz auf verschiedene Anhaltspunkte, wie z. B. der Sprache, die Fähigkeit Gefühle auszudrücken und das Empfinden von Emotionen (Toader et al. 2020). Menschenähnliche Gestaltungselemente (wie Name und Avatar) können bei den Nutzenden eine solche soziale Reaktion auslösen, wie z. B. das Gefühl der Anwesenheit eines Mitarbeiters, welches sich wiederum positiv auf das Kundenerlebnis auswirken kann. Entsprechend kann auch im Kontext einer Reiseimpfungsberatung durch einen Chatbot davon ausgegangen werden, dass menschenähnliche Gestaltungselemente in Nutzer:innen eine Wahrnehmung von sozialer Präsenz herbeiführen werden. Aus diesem Grund leiten wir folgende Hypothese ab:

### H1

Ein menschenähnliches Erscheinungsbild eines Chatbots führt zu einer höheren wahrgenommenen sozialen Präsenz.

Des Weiteren beziehen wir uns in unserer Studie auf das Konzept des Vertrauens. Vertrauen spielt in fast jeder Beziehung eine entscheidende Rolle und ist grundlegend, wenn es z. B. um die Glaubwürdigkeit geht. Insbesondere im medizinischen Kontext gilt, dass Vertrauen die Basis einer effektiven Arzt-Patienten-Beziehung ist. Studien haben gezeigt, dass Vertrauen als messbare Größe mit dem Gesundheitszustand einhergehen und das Misstrauen sich dabei auch negativ auf die Arzt-Patienten-Beziehung auswirken kann (d’Hombres et al. 2010). In unserer Studie beziehen wir uns auf die drei Faktoren, die Vertrauen beeinflussen können: Fähigkeit, Integrität und Wohlwollen (Mayer et al. 1995). Im Kontext von Chatbots kann fähigkeitsbasiertes Vertrauen als die durch die Nutzer:innen wahrgenommenen Fähigkeiten oder Kompetenzen des Chatbots verstanden werden. Unter dem integritätsbasierten Vertrauen wird eine Übereinkunft in den Werten und Prinzipien zwischen Chatbot und Nutzer:in verstanden. Abschließend beschreibt das wohlwollensbasierte Vertrauen, dass der Chatbot keine Ziele verfolgt, die im Widerspruch zu den Zielen und Bedürfnissen des Nutzenden stehen.

Im Zusammenspiel mit sozialer Präsenz und zwischenmenschlichem Vertrauen wurde bereits aufgezeigt, dass diese beiden Faktoren miteinander korrelieren. So konnten Brendel et al. (2022) in ihrer Studie zeigen, dass soziale Präsenz sich im Gesundheitskontext positiv auf das Vertrauen der Nutzer:innen auswirkt. Aus diesen Gründen gehen wir davon aus, dass eine hohe wahrgenommene soziale Präsenz die Vertrauensfaktoren positiv beeinflussen. Daraus leiten wir folgende Hypothese ab:

### H2a

Eine höhere wahrgenommene soziale Präsenz wirkt sich positiv auf die wahrgenommene Fähigkeit aus.

### H2b

Eine höhere wahrgenommene soziale Präsenz wirkt sich positiv auf das wahrgenommene Wohlwollen aus.

### H2c

Eine höhere wahrgenommene soziale Präsenz wirkt sich positiv auf die wahrgenommene Integrität aus.

Darüber hinaus gilt es zu untersuchen, welcher der drei Vertrauenskonstrukte am stärksten das letztendliche Vertrauen der Nutzer:innen in den Chatbot zur Reiseimpfungsberatung beeinflusst. Dazu wurden folgende Hypothesen aufgestellt:

### H3a

Die wahrgenommene Fähigkeit wirkt sich positiv auf das Vertrauen in den Chatbot aus.

### H3b

Wahrgenommenes Wohlwollen wirkt sich positiv auf das Vertrauen in den Chatbot aus.

### H3c

Wahrgenommene Integrität wirkt sich positiv auf das Vertrauen in den Chatbot aus.

Letztendlich muss es das Ziel eines jeden Chatbots sein, die Nutzer:innen zufriedenzustellen. Auch im Kontext der Reiseimpfungsberatung gilt es, dass die Nutzer:innen am Ende mit einem guten Gefühl aus der Beratung herauskommen. Um diese Zufriedenheit mit der Dienstleistung sicher zu stellen, gilt es zu verstehen, wie sich die wahrgenommene soziale Präsenz und das Vertrauen in den Chatbot darauf auswirken. Grundsätzlich sollten beide Faktoren einen positiven Einfluss haben, da auch in der Beratung zwischen Arzt oder Ärztin und Patient:in beide Faktoren wichtig sind (Cukor et al. 1998; Hall et al. 2001). Wenn ein Arzt oder Ärztin als unfreundlich und kühl wahrgenommen wird, fühlen sich viele Patienten und Patientinnen nicht wohl – Ihnen fehlt die soziale Präsenz. Ebenso muss einem Arzt oder Ärztin auch vertraut werden, damit dessen Beratung als zufriedenstellend wahrgenommen wird. Daher wurden die folgenden zwei Hypothesen formuliert:

### H4

Die wahrgenommene soziale Präsenz wirkt sich positiv auf die Servicezufriedenheit aus.

### H5

Das wahrgenommene Vertrauen in den Chatbot wirkt sich positiv auf die Servicezufriedenheit aus.

## Experiment

### Experimentaufbau

Zur Beantwortung der Forschungsfragen wurde ein Online-Experiment mit zwei verschieden gestalteten Chatbots durchgeführt. Die detaillierten Differenzierungen der beiden Implementierungen der Chatbots sind aus der nachfolgenden Tab. 1 zu entnehmen. Grundsätzlich wurde einer der Chatbots mit möglichst vielen menschlichen Elementen ausgestattet, während der andere keine solcher Elemente aufweist. Dabei wurden sowohl verbale (Parasuraman und Miller 2004; Bickmore und Picard 2005; Derrick und Ligon 2014; Feine et al. 2019) als auch non-verbale Gestaltungselemente verwendet (Hoff und Bashir 2015).

**Tab. 1:** Übersicht der Gestaltungselemente im nicht menschlichen vs. menschlichen Chatbot

	Gestaltungselemente	Nicht menschenähnlicher Chatbot	Menschenähnlicher Chatbot
Verbale	*Inhalt*		
	Konversationsfähigkeit	X	X
	Rat & Empfehlung	X	X
	Name	–	X
	Bedanken	–	X
	Begrüßung	–	X
	Loben	–	X
	Selbstauskunft	–	X
	*Style*		
	Formal	X	X
	Proaktive Interaktion	X	X
	Sprache	X	X
	Lexikalische Diversität	–	X
Visuell	*Computer-vermittelte-Kommunikation*		
	Taste/Knopf	X	X
	*Darstellung*		
	Namentag	X	X
	*Erscheinungsbild*		
	2D-Visualisierung	–	X
	Alter	–	X
	Grad der Menschenähnlichkeit	–	X
	Kleidung	–	X
	Titelzeile mit Namen	–	X
Unsichtbare	*Polychronismus*		
	Eröffnungsnachricht	X	X

**Abb. 2:**
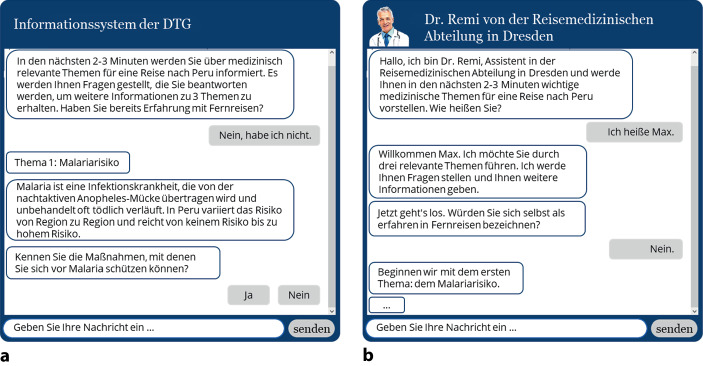
Nicht menschenähnlicher Chatbot (**a**) vs. menschenähnlicher Chatbot (**b**)

Die unterschiedlichen Gestaltungen sind in Abb. 2 veranschaulicht

### Teilnehmende

Die Durchführung der Datenerhebung hat in der Zeit vom 21. Februar bis zum 14. März 2022 stattgefunden. Die Studie war im Internet frei zugänglich und wurde über soziale Netzwerke promotet. Innerhalb dieser 22 Tage nahmen insgesamt 78 Personen erfolgreich an der Studie teil. Von den Teilnehmenden waren 64,1 % (50 Teilnehmerinnen) Frauen und 35,9 % (28 Teilnehmer) waren Männer. Die Altersspanne reichte von 18–74 Jahren (M = 35,55 Jahre, SD = 15,56). Das Experiment wurde in deutscher Sprache mit deutschsprachigen Teilnehmenden durchgeführt. Die Teilnehmer:innen wurden über soziale Medien (Facebook.com, Nebenan.de, SurveyCycle.com) und persönliche Netzwerke rekrutiert. Als Anreiz wurde allen Teilnehmer:innen die Teilnahme an einer Verlosung von fünf 10-Euro-Gutscheinen angeboten.

### Fragebogen

Um die Auswirkungen der Vertrauenswürdigkeit und der Service Zufriedenheit im Zusammenhang mit anthropomorphen Gestaltungselementen zu erforschen, nutzten wir etablierte Fragen aus früheren Studien zur Messung (Mayer et al. 1995; Gefen und Straub 1997; Merritt 2011; Verhagen et al. 2014). Diese sind in nachfolgender Tab. 2 zusammengefasst. Die Messung der Konstrukte basierte auf einer Likertskala, von 1 (stimme voll zu) bis 7 (stimme überhaupt nicht zu). Während der Befragung wurden Kontrollfragen integriert, um die Aufmerksamkeit der Teilnehmer:innen zu überprüfen.

**Tab. 2:** Konstrukte und Faktorladungen (*N* = 78)

Konstrukte	Faktorladungen
*Soziale Präsenz (α* *=* *0,928, CR* *=* *0,949, AVE* *=* *0,823),* (Gefen und Straub 1997)	
[SP1] Ich spürte Menschlichkeit bei der Interaktion mit dem System	0,912
[SP2] Ich spürte Kontaktfreudigkeit bei der Interaktion mit dem System	0,911
[SP3] Ich spürte Zuneigung bei der Interaktion mit dem System	0,910
[SP4] Ich spürte einen menschlichen Spürsinn bei der Interaktion mit dem System	0,935
*Fähigkeit (a* *=* *0,935, CR* *=* *0,958, AVE* *=* *0,884), *(Mayer et al. 1995)	
[ITA1] Das System ist kompetent	0,928
[ITA2] Das System ist in der Lage, angehende Reisende zufriedenzustellen	0,947
[ITA3] Man kann von dem System eine gute Beratung erwarten	0,946
*Wohlwollen (α* *=* *0,817, CR* *=* *0,891, AVE* *=* *0,733)*	
[ITB1] Das System ist wirklich am Wohl der Reisenden interessiert	0,885
[ITB2] Das System stellt die Interessen der Reisenden in den Vordergrund	0,882
[ITB3] Wenn Probleme auftauchen, kann man erwarten, vom System fair behandelt zu werden	0,798
*Integrität (α* *=* *0,890, CR* *=* *0,932, AVE* *=* *0,820)*	
[ITI1] Man kann den Aussagen des Systems Glauben schenken	0,889
[ITI2] Das System arbeitet gewissenhaft	0,926
[ITI3] Ich bin mit den Prinzipien, nach denen das System arbeitet, zufrieden	0,901
*Human-automation Vertrauen (a* *=* *0,934, CR* *=* *0,949, AVE* *=* *0,756),* (Merritt 2011)	
[HAT1] Ich glaube, dass das System eine kompetente Leistung erbringt	0,885
[HAT2] Ich vertraue dem System	0,923
[HAT3] Ich habe Vertrauen in die Beratung durch das System	0,903
[HAT4] Ich kann mich auf das System verlassen	0,916
[HAT5] Ich kann mich darauf verlassen, dass das System widerspruchsfrei ist	0,836
[HAT6] Ich kann mich darauf verlassen, dass das System jedes Mal sein Bestes tut, wenn ich Informationen benötige	0,737
*Service Zufriedenheit (a* *=* *0,915, CR* *=* *0,947, AVE* *=* *0,820),* (Verhagen et al. 2014)	
[S1] Ich bin mit dem Service des Systems zufrieden	0,939
[S2] Ich bin mit der Art und Weise, wie das System mich behandelt hat, zufrieden	0,881
[S3] Ich bin mit der gesamten Interaktion zufrieden	0,953

*α* Cronbach’s Alpha, *CR* Composite Reliability, *AVE* Average Variance Extracted

Hinweis: Alle Konstrukte wurden für die Umfrage ins Deutsche übersetzt

## Ergebnisse

**Abb. 3:**
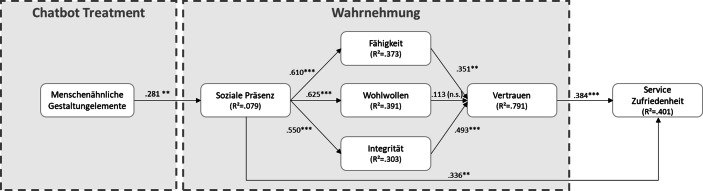
Ergebnisse des Forschungsmodells. *** *=* *p* *<* 0,001, ** *=* *p* *<* 0,01, * *=* *p* *<* *0,05*

Die Ergebnisse zeigen, dass die wahrgenommene soziale Präsenz signifikant die wahrgenommene Vertrauenswürdigkeit und die Zufriedenheit mit dem Service des Chatbots erhöhen. Dabei ist die Verwendung eines Chatbots mit menschenähnlichen Gestaltungselementen von ausschlaggebender Bedeutung. Auch wirkt sich die soziale Präsenz signifikant auf die drei Haupttreiber der Vertrauensbildung aus. Es wird ersichtlich, dass die Integrität sich am stärksten auf das Vertrauen auswirkt, gefolgt von der Fähigkeit bzw. Kompetenz des Systems. Wohlwollen hat keinen signifikanten Einfluss auf das Vertrauen. Die nachfolgende Abb. 3 veranschaulicht die Forschungsergebnisse und zeigt die jeweiligen Signifikanzen und β‑Werte auf.

Des Weiteren wurden unsere abgeleiteten Hypothesen weitestgehend bestätigt. Diese wurden mit der Software SmartPLS (Version 3.3.7) getestet. Das endgültige Modell basierte auf einem Bootstrapping-Resampling-Ansatz mit 5000 Stichproben. Die ermittelten Faktorladungen zeigen eine zuverlässige und gültige Messung. Auch die R^2^-Werte zeigen eine zuverlässige Wirkung. Während für Fähigkeit, Wohlwollen, Integrität, Vertrauen und Service Zufriedenheit die Wirkung hoch ist (alle > 0,25), weißt die soziale Präsenz eine geringe Korrelation auf (< 0,13) (Cohen 2013).

Insgesamt konnten acht der neun Hypothesen bestätigt werden (siehe Tab. 3). Die Studie bestätigt, dass soziale Präsenz Vertrauen und Service Zufriedenheit stark positiv beeinflusst. Für die Hypothese, dass sich Wohlwollen positiv auf das Vertrauen auswirkt, wurde keine Signifikanz festgestellt.

**Tab. 3:** Ergebnisse

Hyp	Beziehung	β‑Wert	t‑Wert	*p*-Wert	Bestätigung
H1	Menschenähnliche Gestaltung → Soziale Präsenz	.281	2.707	.007**	*Unterstützt*
H2a	Soziale Präsenz → Fähigkeit	.610	9.167	.000***	*Unterstützt*
H2b	Soziale Präsenz → Wohlwollen	.625	8.479	.000***	*Unterstützt*
H2c	Soziale Präsenz → Integrität	.550	6.344	.000***	*Unterstützt*
H3a	Fähigkeit → Vertrauen	.351	2.669	.008**	*Unterstützt*
H3b	Wohlwollen → Vertrauen	.113	1.274	.203	*Nicht unterstützt*
H3c	Integrität → Vertrauen	.493	5.384	.000***	*Unterstützt*
H4	Soziale Präsenz → Service Zufriedenheit	.336	2.743	.006**	*Unterstützt*
H5	Vertrauen → Service Zufriedenheit	.384	3.390	0.001**	*Unterstützt*

Alle β‑Werte sind standardisiert. | *** *=* *p* *<* 0,001, ** *=* *p* *<* 0,01, * *=* *p* *<* *0,05*

## Diskussion

Unsere Ergebnisse zeigen dass soziale Präsenz einen signifikanten positiven Einfluss auf alle der drei vertrauenserzeugende Konstrukte hat und sich ebenfalls positiv auf die Service Zufriedenheit auswirkt. Von den drei betrachteten vertrauenserzeugenden Faktoren wirken Fähigkeit und Integrität auf das Vertrauen der Nutzer:innen in den Chatbot. Vertrauen hat seinerseits einen hohen Einfluss auf die Service Zufriedenheit des Nutzers oder der Nutzerin, ebenso die soziale Präsenz.

Von einem praktischen Standpunkt aus zeigt unsere Studie, dass Nutzer:innen einem menschlich gestalteten Chatbot vertrauen und mit dem Service zufrieden sind. Obwohl es sich hierbei um ein Bereich handelt, welcher für die Nutzer:innen ein gewisses Risiko darstellt, da eine Fehlberatung gesundheitliche Konsequenzen mit sich bringt. Trotzdem reagierten die Nutzer:innen durchweg positiv auf den Chatbot. Dies zeigt dass Chatbots problemlos im Bereich der Digitalen Gesundheitsdienste von Nutzern und Nutzerinnen akzeptiert werden.

In dieser Studie hat sich Vertrauen als wichtiger Einfluss auf die Service Zufriedenheit ergeben. Dieser Effekt lässt sich durch den Gesundheitskontext erklären, welcher eine starke Vertrauensbasis zwischen den Patienten und Patientinnen und den Dienstleistungsanbieter benötigt. Es ist davon auszugehen, dass dieser Effekt auch auf andere IT-Dienstleistungen im Gesundheitssektor übertragbar ist. Bei der Gestaltung und Evaluation solcher IT-Dienstleistungen sollte vermehrt Augenmerk auf das Vertrauen der Nutzenden gerichtet werden. Beispiele für solche vertrauensfördernden Herangehensweisen sind: die Funktionsweise der IT-Dienstleistung für den Nutzer transparent gemacht werden (z. B. via explainable AI, Khurana et al. 2021), oder dass die Patienten oder Patientinnen einen einfachen Zugriff auf ihre Gesundheitsdaten erlangen können (patient empowerment).

Die Ergebnisse der Studie unterstreichen die Bedeutung für die Verwendung von menschenähnlichen Gestaltungselementen in Chatbots. Mittels dieser Gestaltungselemente kann sowohl die Service Zufriedenheit als auch das Vertrauen des Nutzers oder der Nutzerin erhöht werden. Dies ist vor allem relevant für Kontexte, in welchen Nutzervertrauen eine große Rolle spielt, wie z. B. Gesundheitswesen, Bankwesen oder der Versicherungsbranche. Entscheidend hierbei ist, dass das Vertrauen und die Nutzerzufriedenheit auch im Onlinekontext durch einfache Gestaltungselemente erreicht werden kann. In dieser Studie wurden entsprechende Gestaltungselemente identifiziert und auf ihre Wirkung auf die Nutzer:innen untersucht.

Die korrekte Wahl der Gestaltungselemente wirken sich maßgeblich auf das Vertrauen und die Service Zufriedenheit der Nutzer:innen in den Chatbots aus. Eine fundierte Wahl der Gestaltungselemente stellt eine erhöhte Service Zufriedenheit und Vertrauen der Nutzer:innen sicher. Unternehmen in den obenan genannten Bereichen sind dementsprechend angehalten, entsprechende Gestaltungselemente erst nach einer ausführlichen Evaluation zu verwenden.

Ein interessantes Ergebnis unserer Studie ist, dass sich Wohlwollen nicht auf das Vertrauen auswirkt. Eine mögliche Erklärung ist, dass die Nutzer:innen den Chatbot nicht als Entität mit eigenen Zielen wahrnehmen. Vereinfacht ausgedrückt, die Nutzer:innen glauben nicht, dass der Chatbot andere Ziele als seine offensichtlich dargestellten verfolgen kann. Dies beachtet allerdings nicht, dass hinter dem Chatbot ein:e Programmierer:in oder Unternehmen steht, welches durchaus seine eigenen Ziele verfolgt, die nicht mit den Zielen des Nutzers oder der Nutzerin kompatibel sein müssen. Hier ist ein Versagen der Theory of Mind sichtbar, also der Erkenntnis, das andere Entitäten ihre eigenen Ziele verfolgen und damit eine Gelegenheit für unethische Manipulation des Nutzers oder der Nutzerin. Diese These eröffnet interessante Forschungsfragen für zukünftige Untersuchungen.

Unsere Forschung trägt zur Verbesserung der Mensch-Computer-Interaktion im Kontext der Chatbot-Gestaltung bei. Unsere Studie unterstützt bestehende Forschungsarbeiten zum positiven Einfluss von menschenähnlichen Gestaltungselementen, wahrgenommener sozialer Präsenz, wahrgenommener Menschlichkeit und Servicequalität. Diese Forschung haben wir um den Kontext digitale Gesundheit und reisemedizinische Aufklärung erweitert.

## Limitation

Unsere Studie wurde mit allem nötigen Bedacht und nach wissenschaftlichen Standards durchgeführt. Trotzdem ergeben sich eine Reihe von Punkten, welche in der Bewertung beachtet werden sollten.

Die Studie wurde nur mit deutschen Teilnehmenden durchgeführt. Aufgrund der kulturellen Unterschiede in der Wahrnehmung von sozialer Präsenz (Hassanein et al. 2009) sind unsere Ergebnisse nicht auf andere Kulturkreise übertragbar. Ebenfalls fand keine Beratung im Sinne eines Dialoges mit den Nutzern und Nutzerinnen in Bezug auf die eigene persönliche Situation und besonderen Ansprüche statt. Es wurden die generellen reisemedizinischen Informationen in Bezug zu einer hypothetischen Reise nach Peru vermittelt.

Eine weitere Limitierung unserer Studie ist die Verwendung von Fragebögen, um unterbewusste und automatische Eindrücke wie soziale Präsenz und Vertrauen zu erfassen. Fragebögen beruhen auf einer bewussten Reflexion der Situation und leiden unter einer Reihe von Verzerrungen wie soziale Erwünschtheit (die Teilnehmenden geben die Antwort, die sie für sozial angemessen halten), Tendenz zur Mitte (Teilnehmende wählen eher einen der mittleren Punkte aus) oder Akquieszenz (Teilnehmende tendieren dazu, zuzustimmen). Eine Möglichkeit diese Verzerrungen zu vermeiden ist, die Fragebögen durch physiologische Messungen zu ergänzen. Für beide Konstrukte wurden spezifisch messbare Gehirnaktivitäten beschrieben, welche eine unterstützende Erfassung erlauben würden (Dimoka 2010; Soiné et al. 2021).

Unsere Studie ist in hohem Maße von der Auswahl der verwendeten menschenähnlichen Gestaltungselemente und Kommunikationsstrukturen abhängig. Eine Auswahl anderer Gestaltungselemente könnte zu einem anderen Empfinden bei den Nutzern und Nutzerinnen führen, vor allem aufgrund der Kontextabhängigkeit der untersuchten Effekte. Es bedarf weitere Forschung, um weitere Gestaltungselemente und Strukturen zu untersuchen.

## Fazit

Unsere Forschungsergebnisse erweitern das Wissen im Bereich der Mensch-Computer-Interaktion in Bezug auf die vertrauensvolle Interaktion von Menschen und Chatbots im Gesundheitskontext. Konkret wurde untersucht, wie sich die menschähnliche Gestaltung von Chatbots auf die Wahrnehmung der Nutzer:innen im Kontext der reisemedizinischen Aufklärung auswirkt. Unsere Ergebnisse zeigen, dass sich soziale Präsenz auf die wahrgenommene Vertrauenswürdigkeit und die Service Zufriedenheit auswirkt. Chatbots mit menschenähnlichen Gestaltungselementen erhöhen das Vertrauen der Nutzer:innen signifikant. Entsprechend ist die menschenähnliche Gestaltung von Chatbots ein positiver Faktor für die effektive Aufklärung im reisemedizinischen Kontext. Unsere Forschungsergebnisse zeigen, dass die kontextangemessene Auswahl von menschenähnlichen Gestaltungselementen maßgeblich das Vertrauen und die Service Zufriedenheit der Nutzer:innen beeinflusst.
